# Autoimmune astrocytopathy double negative for AQP4‐IgG and GFAP‐IgG: Retrospective research of clinical practice, biomarkers, and pathology

**DOI:** 10.1111/cns.70042

**Published:** 2024-09-15

**Authors:** Pei‐Hao Lin, Hai‐Yan Yao, Li Huang, Cong‐Cong Fu, Xiao‐Li Yao, Chun Lian, Shi‐Feng Zhang, Wen‐Dong Lai, Guan‐Yan Lin, Sha Liao, Jie Yang, Zhi‐Feng Mao, Ding Liu, Bao‐Yi Long, Jia‐Jia Yue, Cong Gao, You‐Ming Long

**Affiliations:** ^1^ Key Laboratory of Neurogenetics and Channelopathies of Guangdong Province, Department of Neurology, Institute of Neuroscience, Ministry of Education of China, The Second Affiliated Hospital Guangzhou Medical University Guangzhou China; ^2^ Department of Neurology, Guangzhou Eighth People's Hospital Guangzhou Medical University Guangzhou China; ^3^ Department of Neurology The First Affiliated Hospital of Sun Yat‐sen University Guangzhou China; ^4^ Institution of Kingmed Guangzhou Medical University Guangzhou China; ^5^ Department of Neurology The Third Xiangya Hospital of Central South University Changsha China; ^6^ The First Clinical College Changsha Medical University Changsha China

**Keywords:** autoimmune astrocytopathy, autoimmune encephalitis, glial fibrillary acidic protein, neurofilament light chain

## Abstract

**Objective:**

The objective of this study is to investigate the presence of astrocyte antibodies in patients, excluding aquaporin‐4 or glial fibrillary acidic protein (GFAP) antibodies, while evaluating associated biomarkers and pathologies.

**Methods:**

Patient serum and cerebrospinal fluid (CSF) were tested for antibodies using tissue‐ and cell‐based assays. Neurofilament light chain (NFL) and GFAP in the CSF were detected using single‐molecule array (SIMOA).

**Results:**

116 patients accepted SIMOA. Fifteen functional neurological disorders patients without antibodies were designated as controls. Thirty‐five patients were positive for astrocyte antibodies (Anti‐GFAP: 7; Anti‐AQP4: 7; unknown antibodies: 21, designed as the double‐negative group, DNAP). The most frequent phenotype of DNAP was encephalitis (42.9%), followed by myelitis (23.8%), movement disorders (19.0%), and amyotrophic lateral sclerosis‐like (ALS‐like) disease (14.2%). The levels of CSF GFAP and NFL in DNAP were higher than in the control (GFAP: 1967.29 [776.60–13214.47] vs 475.38 [16.80–943.60] pg/mL, *p* < 0.001; NFL: 549.11 [162.08–2462.61] vs 214.18 [81.60–349.60] pg/mL, *p* = 0.002). GFAP levels decreased in DNAP (*n* = 5) after immunotherapy (2446.75 [1583.45–6277.33] vs 1380.46 [272.16–2005.80] pg/mL, *p* = 0.043), while there was no difference in NFL levels (2273.78 [162.08–2462.61] vs 890.42 [645.06–3168.06] pg/mL, *p* = 0.893). Two brain biopsy patterns were observed: one exhibited prominent tissue proliferation and hypertrophic astrocytes, with local loss of astrocytes, while the other showed severe astrocyte depletion with loss of neurofilaments around the vessels. Eighteen patients received immunotherapy, and improved except one with ALS‐like symptoms. We identified anti‐vimentin in this patient.

**Discussion:**

There are unidentified astrocyte antibodies. The manifestations of double‐negativity are heterogeneous; nevertheless, the pathology and biomarkers remain consistent with astrocytopathy. Immunotherapy is effective.

## Graphical abstract

Current knowledge suggests that autoimmune astrocytopathy primarily involves antibodies targeting AQP4 and GFAP. This study aimed to investigate a novel type of autoimmune astrocytopathy that is negative for both AQP4‐IgG and GFAP‐IgG and explore its clinical manifestations, biomarkers, and pathology.

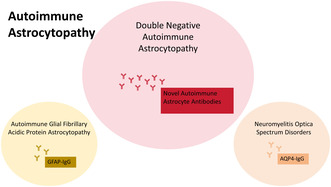

## 1 INTRODUCTION

The concept of autoimmune astrocytopathy originated from a pathological review of neuromyelitis optica spectrum disorders (NMOSD).^1^ Injury and degeneration of astrocytes beyond lesioned tissues, as well as elevated biomarkers of astrocytes, such as glial fibrillary acidic protein (GFAP), reveal the nature of autoimmune astrocytopathy.^1^, ^2^, ^3^ Autoimmune glial fibrillary acidic protein astrocytopathy (GFAP‐A), characterized by similar astrocytic changes and biomarkers, is an astrocytopathy induced by CD8+ T cells and presents as meningoencephalomyelitis.^4^, ^5^ Paraneoplastic neurological syndrome, with positive SOX1 antibody targeting Bergmann glial cells, presents another type of astrocytopathy; however, supporting biomarkers or pathological evidence confirming astrocyte injury remains scarce.^6^, ^7^ Neurons have been shown to have many antigens that can become targets of autoimmune reactions under certain conditions. Similarly, astrocytes may also possess autoantigens beyond just GFAP and aquaporin‐4 (AQP4). Nevertheless, limited knowledge exists regarding associated autoantibodies, clinical phenotypes, and etiopathogenesis. In our previous studies, numerous patients tested positive for astrocyte antibodies through a tissue‐based assay (TBA) with some exhibiting negativity for AQP4‐IgG and GFAP‐IgG via cell‐based assay (CBA).^8^, ^9^

GFAP, an intermediate filament protein localized in the cytoplasm of astrocyte, is considered as a good biomarker of disease activity in astrocytopathy because there is no actively secreted form of the molecule.^10^, ^11^ The GFAP level in the cerebrospinal fluid (CSF) during NMOSD relapses were markedly high and swiftly returned to normal after immunotherapy. In acute phase of NMOSD, CSF levels of GFAP, S100B protein, or myelin basic protein (MBP) shows a strong correlation with the Expanded Disability Status Scale (EDSS) score, while, at the 6‐month follow‐up, only CSF‐GFAP maintains a correlation with EDSS. Because GFAP is highly specific to astrocytes, whereas S100B is also produced by microglia and oligodendrocytes. Serum GFAP levels are also increased in astrocytopathy, but the differences are less significant than those in CSF.^12^, ^13^ Neurofilament light chain (NFL) is exclusively produced by neurons and stands as the most prevalent subunit and critical component of neurofilaments, which are the primary structural elements of the axonal cytoskeleton.^14^, ^15^ Upon axonal damage, NFL is discharged into the extracellular fluid. Studies have revealed that NFL levels in NMOSD patients are substantially higher than those in healthy control groups.^16^, ^17^, ^18^ This finding substantiates the presence of axonal injury in individuals with NMO. Therefore, in this study, we used GFAP and NFL as biomarkers of astrocytes and neurons, respectively.

To date, no reports have delineated the clinical phenotype, treatment, and outcome of such cases, nor have studies confirmed astrocytopathy using biomarkers and pathology. Hence, we retrospectively analyzed the clinical data, biomarkers in CSF, and pathology of patients with astrocyte antibodies to confirm the presence of autoimmune astrocytopathy beyond AQP4 and GFAP autoimmunity and provide new insights into diagnosis and therapy.

## 2 METHODS

**FIGURE 1:**
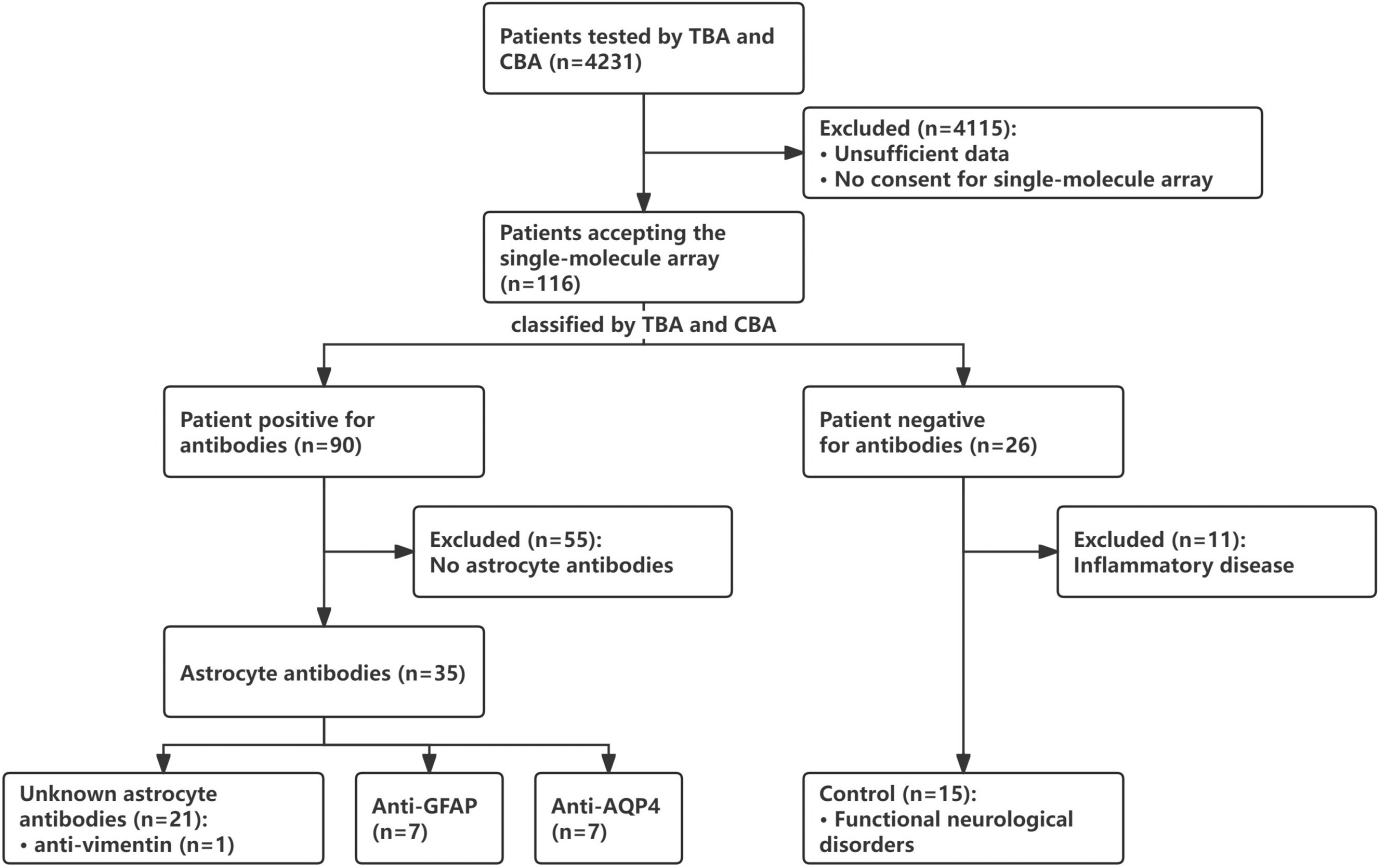
Flow chart of grouping. Among 4241 patients underwent TBA and CBA testing, 116 patients accepted the single‐molecule array. Of these, both serum and CSF of 26 patients tested negative, while 90 patients tested positive in either serum or CSF. Among the antibody‐negative patients, 15 with functional neurological disorders, instead of CNS inflammatory diseases, served as the control group in the present study. Among the positive cases, 35 (38.9%, 35/90) were positive for astrocytes, and 55 (61.1%, 55/90) were not positive for astrocytes but neurons or oligodendrocytes. Based on clinical data and CBA results, seven (20.0%, 7/35) with AQP4‐IgG (six detected in serum and one in both serum and CSF) were classified as NMOSD group, seven (20.0%) with GFAP‐IgG (all detected in only CSF) were defined as GFAP‐A group, and 21 (60.0%, 21/35) were negative for AQP4‐IgG and GFAP‐IgG by CBA were classified as double negative astrocytopathy (DNAP) group (seven in serum, three in CSF, 11 in both serum and CSF). In DNAP, a patient was positive for anti‐vimentin. The range of CBA included GFAP‐IgG, AQP4‐IgG, MOG‐IgG, MBP‐IgG, IgLON5‐IgG, DPPX6‐IgG, GlyR1‐IgG, DRD2‐IgG, GAD65‐IgG, mGluR5‐IgG, NMDAR‐IgG, AMPA1‐IgG, AMPA2‐IgG, LGI 1‐IgG, CASPR2‐IgG, GABAB‐IgG, mGluR1‐IgG, and Antisynaptin‐3 αIgG.

From April 2021 to June 2023, 4241 patients underwent TBA and CBA testing at the Second Affiliated Hospital of Guangzhou Medical University. Among them, 116 patients with 133 CSF samples (116 acquired before treatments and 17 acquired after treatments) accepting the single‐molecule array (SIMOA) were included in the study (Figure 1).

### 2.1 Tissue‐based assay

Every CSF (1:1) or serum (1:150) dilution underwent a 2‐h incubation at room temperature, while reacting with monkey cerebellar and hippocampus substrates on glass slides using a TBA kit (BioSystems). Following incubation, the slides were washed twice with phosphate‐buffered saline (PBS) and incubated with fluorescein‐conjugated goat anti‐human IgG for 30 min. Subsequently, the slides were then rinsed with PBS, and the resulting fluorescence patterns were examined under a microscope and judged independently by two researchers (You‐Ming Long and Pei‐Hao Lin). The pattern of astrocytic antibodies was defined in our previous study.^19^

### 2.2 Cell‐based assay

Conducted by Guangzhou KingMed Center for Clinical Laboratory Co., Ltd, the samples were detected for GFAP‐IgG, AQP4‐IgG and others, including MOG‐IgG, MBP‐IgG, IgLON5‐IgG, DPPX6‐IgG, GlyR1‐IgG, DRD2‐IgG, GAD65‐IgG, mGluR5‐IgG, NMDAR‐IgG, AMPA1‐IgG, AMPA2‐IgG, LGI 1‐IgG, CASPR2‐IgG, GABAB‐IgG, mGluR1‐IgG, and Antisynaptin‐3 αIgG. Detection for GFAP‐IgG and AQP4‐IgG were repeated by in‐house CBA at the Second Affiliated Hospital of Guangzhou Medical University.

### 2.3 Single‐molecule Array

SIMOA was conducted by the Guangzhou KingMed Center for Clinical Laboratory Co., Ltd. to detect neurofilament light chain (NFL) and GFAP levels in CSF samples. SIMOA has been demonstrated to be more sensitive and effective than common chemiluminescence assay or Enzyme‐Linked Immunosorbent Assay (ELISA).^20^, ^21^, ^22^ Its application for testing NFL and GFAP has been validated in previous studies.^11^, ^21^, ^22^, ^23^

### 2.4 Pathology

Brain tissue pathology was conducted at The Second Affiliated Hospital of Guangzhou Medical University, as described in our previous studies.^19^ Tissue slices (10‐μm thick) were meticulously cut and sequentially placed onto numbered slides, facilitating the comparison of molecular distribution across adjacent serial sections. Hematoxylin and eosin (H&E) staining, along with routine immunohistochemical procedures, was performed at our hospital. Peripheral nerve and muscle pathology analyses were conducted at the Guangzhou KingMed Center for Clinical Laboratory Co., Ltd.

### 2.5 Construction of vimentin plasmids and antibody detection

HEK293T cells were transfected with a human‐targeting cDNA plasmid (NM_003380.5). 36 h after transfection, the cells were fixed with 4% paraformaldehyde and then incubated with serum (1:10) or CSF (1:1) from a TBA‐positive patient for 1 h at room temperature. Subsequently, the cells were incubated for another hour with the corresponding fluorescent secondary antibodies (1:1000 dilution; CY3‐conjugated goat anti‐human IgG; Bioss). Images were captured using a fluorescence microscope.

### 2.6 Western blot analysis

#### 2.6.1 Anti‐vimentin autoantibody identification

U‐251 MG cells were collected and lysed in radioimmunoprecipitation assay (RIPA) buffer supplemented with protease and phosphatase inhibitors (Yeasen Biotechnology). The extracted proteins were mixed with 5× loading buffer, boiled for 4 min at 100°C, and separated by 10% sodium dodecyl sulfate‐polyacrylamide gel electrophoresis. Then, 30 μg of extracted total protein was transferred to polyvinylidene fluoride (PVDF) membranes (Merck Millipore). The membranes were blocked with 5% skim milk in phosphate‐buffered saline‐Tween 20 (PBST) for 1 h at room temperature and then incubated with the patients' sera at a dilution of 1:1000 or with antibody specific to human vimentin (1:2000, Bioss) at 4°C overnight. Thereafter, the membranes were washed with PBST and incubated with horseradish peroxidase (HRP)‐conjugated anti‐human IgG secondary antibody (1:8000, Yeasen Biotechnology) for another hour at room temperature. Chemiluminescence was used for visualization (PerkinElmer), while FluorChemTM SP software was used to measure the band intensity.

#### 2.6.2 Immunodepletion experiments

For immunodepletion experiments, the patient serum (1:1000 dilution) was incubated with the PVDF membrane containing 2 μg of blotted recombinant human vimentin protein (RayBiotech Life, Inc.) at 37°C for 2 h. Then, it was incubated with a PVDF membrane containing 30 μg of blotted protein extracted from U‐251 MG cells at 4°C overnight. Subsequently, the membranes were incubated with a peroxidase‐conjugated secondary antibody (1:8000, Yeasen Biotechnology) and visualized using chemiluminescence (PerkinElmer).

### 2.7 Statistical analysis

All statistical analyses were conducted using the Statistical Program for Social Sciences software (version 27.0; SPSS). Kolmogorov–Smirnov‐Lilliefors test was used for assessing normality. We applied Bonferroni correction when conducting comparisons across multiple groups. Fisher's exact test was applied for binary and categorical data, while one‐way ANOVA was used for normally distributed data and Kruskal–Wallis tests for non‐normally distributed data. Statistical significance was set at *p* < 0.05 (two‐sided).

### 2.8 Ethics approval

The study protocol (No. 2022‐hs‐56‐02) was approved by the Ethics Committee of the Second Affiliated Hospital of Guangzhou Medical University (Guangzhou China). Participants gave informed consent to participate in the study before taking part.

## 3 RESULTS

### 3.1 Detection results of antibodies and grouping

Grouping was based on the results of TBA and CBA (Figure 1). CSF and serum samples from 116 patients were tested using TBA and CBA. Of these, both serum and CSF of 26 patients tested negative, whereas 90 patients tested positive in either serum or CSF. Among the antibody‐negative patients, 15 with functional neurological disorders, instead of CNS inflammatory diseases (*n* = 11), served as the control group in the present study. Among the positive cases, by TBA, 35 (38.9%, 35/90) were positive for astrocytes, and 55 (61.1%, 55/90) were positive for neurons or oligodendrocytes without stained astrocytes. Patients with astrocyte antibodies were negative for MOG‐IgG, MBP‐IgG, IgLON5‐IgG, DPPX6‐IgG, GlyR1‐IgG, DRD2‐IgG, GAD65‐IgG, mGluR5‐IgG, NMDAR‐IgG, AMPA1‐IgG, AMPA2‐IgG, LGI 1‐IgG, CASPR2‐IgG, GABAB‐IgG, mGluR1‐IgG, and Antisynaptin‐3 αIgG. Among them, seven (20.0%, 7/35) with AQP4‐IgG (six detected in serum and one in both serum and CSF) were classified as NMOSD group, seven (20.0%) with GFAP‐IgG (all detected in only CSF) were defined as GFAP‐A group, and 21 (60.0%, 21/35) were negative for AQP4‐IgG and GFAP‐IgG by CBA were classified as double negative astrocytopathy (DNAP) group (seven in serum, three in CSF, and 11 in both serum and CSF). In the DNAP group, TBA revealed stained astrocytes, stained astrocytes plus neurofilament, and stained astrocytes plus neuron in 14, 3, and 4 patients, respectively.

**FIGURE 2:**
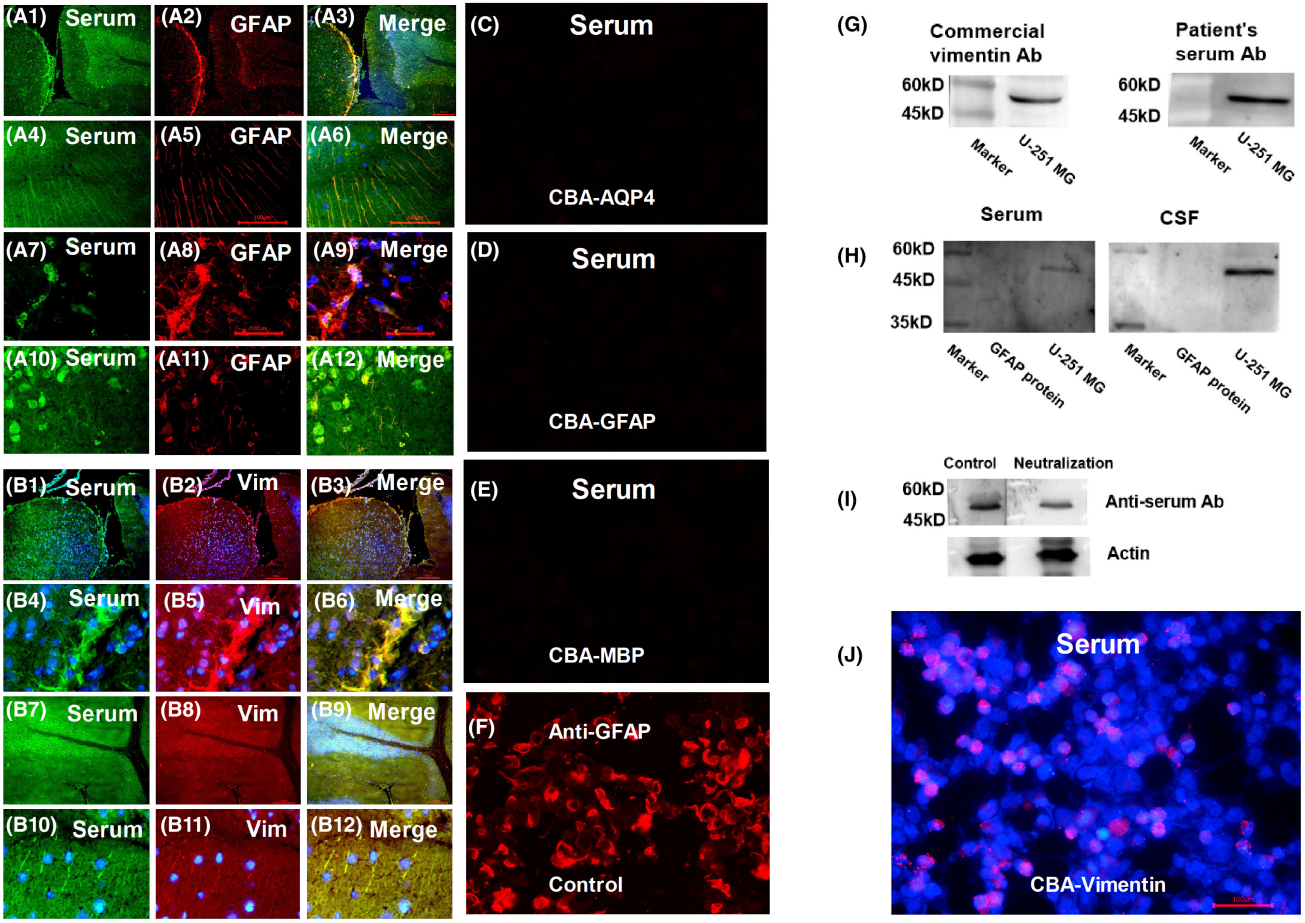
Immunofluorescence pattern of patient IgG bound to the monkey cerebellum, midbrain, and hippocampus in Case 17 and autoantigen identification. (A1–A3) Distribution of serum IgG (green) in the monkey pia or subpia is consistent with astrocytes (staining with GFAP), and IgG of the patient is also stained in neurons (green). (A4–A6) Similar to GFAP‐IgG, the IgG of the patient is reactive with the radial processes of cerebellar cortical Bergmann glia. (A7–A9) However, unlike GFAP‐IgG, the patient's IgG reacts with the inner Virchow–Robin space. (A10–A12) IgG of the patient IgG is largely reactive to hippocampal neurons (green). (B1–B6) Patient IgG (green) and commercial vimentin‐specific IgG (Vim, red) colocalize extensively in the pia, subpia, and neurons (B3). The enlarged image shows the colocalization of patient IgG (green) and commercial vimentin‐specific IgG (red) on the pia mater (B4–B6). (B7–B9) Patient IgG (green) and commercial vimentin–specific IgG (Vim, red) co‐localize extensively in astrocytes and neurons in the cerebellum. (B10–B12) To vimentin, patient IgG reacts with the radial processes of cerebellar cortical Bergmann glia. (C–E) A cell‐based assay (CBA) shows a serum‐negative response to AQP4‐IgG, GFAP‐IgG, and myelin basic protein‐IgG. (F) Positive GFAP‐IgG control is shown. (G–J) Autoantigen for vimentin identification. (G) Western blot analysis showing vimentin protein expression in U‐251 MG cells using a commercial anti‐vimentin primary antibody (left). Western blot analysis showing vimentin protein expression in U‐251 MG cells using the patient's serum as the primary antibody (right). (H) Western blot analysis showing that the patient's serum and CSF reacts to vimentin protein (U‐251 MG cell lysis) but not to recombinant GFAP protein. (I) Vimentin protein expression in U‐251 MG cells decreases after preincubation of the patient's serum with PVDF membrane transferred with 2 μg of recombined human vimentin protein. (J) CBA shows a positive serum response in vimentin‐transinfected HEK‐293 cells. Figure S3 is a higher magnification to reveal a staining pattern that patient IgG and commercial vimentin‐IgG colocalize in neurons and astrocytes.

Additionally, we identified a new antibody against vimentin in the CSF and serum of one patient in DNAP group (Figure 2, Case 17) using fluorescence double labeling, western blotting, competitive inhibition assay, and CBA. The target antigens of astrocyte IgG in other patients remain unconfirmed.

### 3.2 Case description

The case with anti‐vimentin was described as follow. A 51‐year‐old female presented with progressive limbs weakness and atrophy. The symptoms initially started in her left lower limb and subsequently affected her right lower limb and both upper limbs. At admission, her muscle strength was graded as follows: left lower limb 1/5, right lower limb 3/5, left upper limb 4/5, and right upper limb 5−/5. Routine examination of CSF and serum showed no abnormalities. Brain and spinal cord MRI were unremarkable. Electromyography (EMG) revealed widespread motor neuron damage. TBA revealed a stained pattern of neurons and astrocytes in both CSF and serum. GFAP‐IgG, AQP4‐IgG, and others, including MOG‐IgG, MBP‐IgG, IgLON5‐IgG, DPPX6‐IgG, GlyR1‐IgG, DRD2‐IgG, GAD65‐IgG, mGluR5‐IgG, NMDAR‐IgG, AMPA1‐IgG, AMPA2‐IgG, LGI 1‐IgG, CASPR2‐IgG, GABAB‐IgG, mGluR1‐IgG, and Antisynaptin‐3 αIgG were absent from the serum and CSF. CSF levels of GFAP and NFL were measured at 1930 and 1667 pg/mL, respectively. The patient was treated with oral steroids, intravenous methylprednisolone (IVMP), intravenous immunoglobulin (IVIG), rituximab, and immunoadsorption. Despite these interventions, there was no significant improvement in her condition, although her symptoms did not worsen further.

### 3.3 GFAP and NFL levels in CSF among NMOSD, GFAP‐A, and DNAP groups (Figure 3)

**FIGURE 3:**
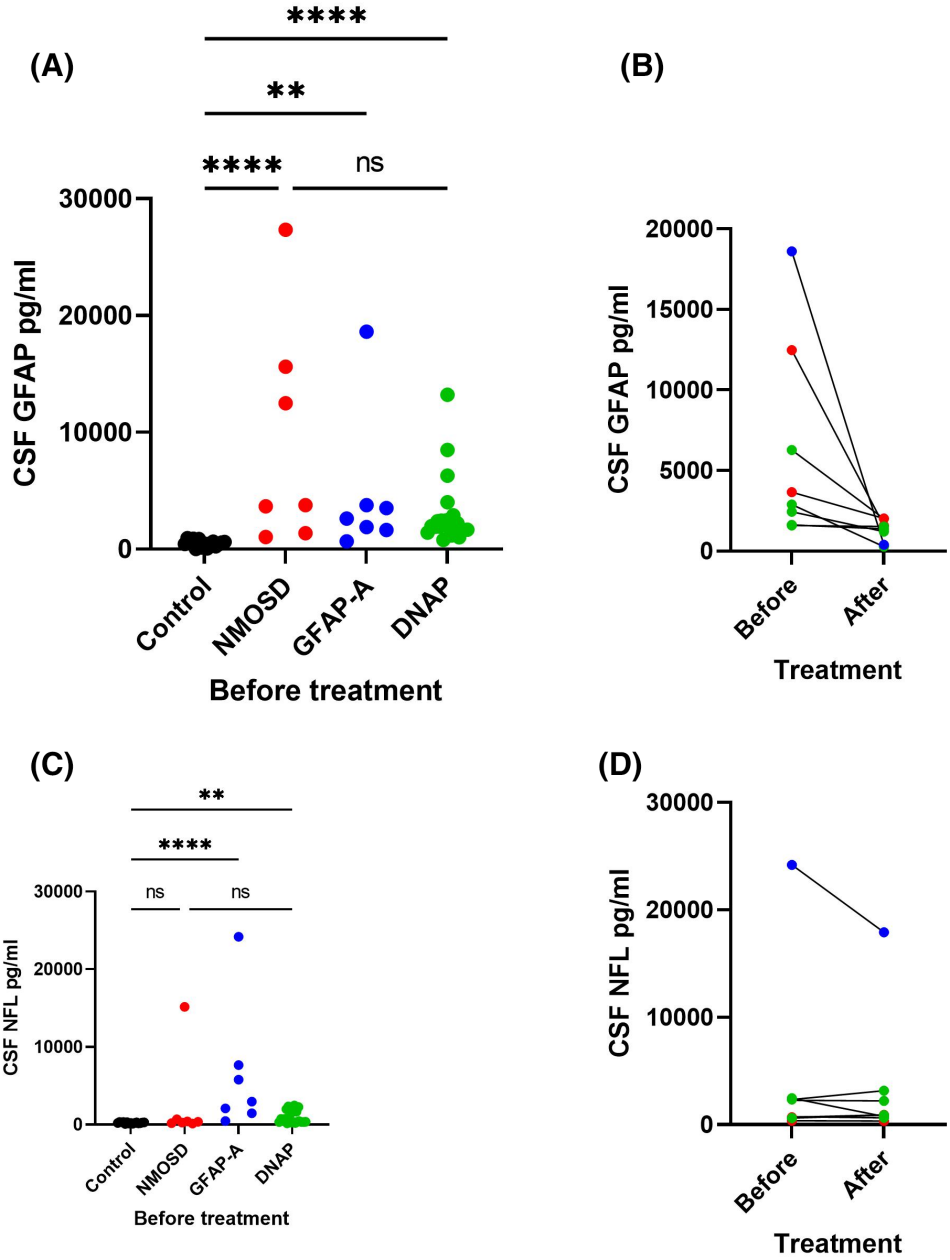
Detection of neurofilament light chain and GFAP in CSF by Single Molecular Array. (A) (Control) Fifteen patients with neurofunctional diseases are included. (NMOSD) Seven patients diagnosed with neuromyelitis optica spectrum disorder with AQP4‐IgG positivity are included. (GFAP‐A) Seven patients diagnosed with GFAP astrocytopathy and who tested positive for GFAP‐IgG are included. (DNAP) 21 patients positive for astrocyte antibodies in the serum or CSF but negative for AQP4‐IgG and GFAP‐IgG are included. (A) Compared with the control, the NMOSD, GFAP‐A, and DNAP groups show higher levels of GFAP in the CSF (*p* < 0.0001, *p* = 0.0014, and *p* < 0.0001, respectively), with no difference among the three groups. (B) Data from five, two, and one patient from the DNAP, NMOSD, and GFAP‐A groups, respectively, before or after treatment. GFAP levels decrease in the DNAP group after treatment (Wilcoxon signed‐rank test; *p* = 0.043). (C) Compared with the control group, the GFAP‐A and DNAP groups show higher levels of neurofilament light chain in the CSF (*p* < 0.0001 and *p* = 0.0019, respectively), with no difference among the three groups. (D) There are no significant differences in patients from the DNAP group before and after treatment. Blue: GFAP‐A; Red: NMOSD; Green: DNAP. The average interval between the collection of CSF samples before and after treatment is 64.4 (±6.5) days. **p < 0.01 ****p < 0.0001.

Neurofilament light chain and GFAP levels in the CSF of patients were analyzed by SIMOA. In the control group, the median GFAP protein levels were 475.38 (16.80–943.60) pg/mL, and NFL protein levels were 214.18 (81.60–349.60) pg/mL.

Glial fibrillary acidic protein levels were significantly higher in the NMOSD, GFAP‐A, and DNAP groups than in the control group with levels of 3773.09 (1047.31–27332.58) pg/mL (*p* < 0.001), 2623.44 (664.96–18600.91) pg/mL (*p* = 0.001), and 1967.29 (776.60–13214.47) pg/mL (*p* < 0.001), respectively, before treatment. There were no statistically significant differences in GFAP levels among the three groups. However, it was observed that GFAP levels decreased in DNAP patients after treatment (2446.75 [1583.45–6277.33] vs 1380.46 [272.16–2005.80] pg/mL, Wilcoxon signed‐rank test, *p* = 0.043). The average interval between the collection of CSF samples before and after treatment is 64.4 (±6.5) days.

Before treatment, the median CSF NFL levels in the NMOSD, GFAP‐A, and DNAP groups were 414.10 (139.81–15170.69), 2958.64 (459.23–24192.47) pg/mL, and 549.11 (162.08–2462.61) pg/mL, respectively. There were no statistically significant differences between the NMOSD and control groups (*p* = 0.568), while significant differences were observed between the GFAP‐A and DNAP groups compared with the control group (GFAP‐A vs. control, *p* < 0.001; DNAP vs. control, *p* = 0.002). No significant differences were found among the three groups. Moreover, there was no significant difference in the NFL levels of patients with DNAP after treatment (2273.78 [162.08–2462.61] vs. 890.42 [645.06–318.06] pg/mL, *p* = 0.893).

### 3.4 Clinical characteristics of patients with DNAP

Table S1 shows the analysis of the 21 patients with DNAP and their valid data. The male/female ratio was 10:11, with a mean (±SD) onset age of 52 (±21.3) years. Various clinical phenotypes in all 21 cases were observed, including nine patients presenting with encephalitis (42.9%, one combined with peripheral neuropathy, one with myelitis and one involving brainstem), five with myelitis (23.8%, one combined with optic nerve and peripheral neuropathy), four with movement disorders (19.0%), three with amyotrophic lateral sclerosis‐like (ALS‐like) disease (14.2%), three with peripheral neuropathy (14.2%), and one with ataxia. Six patients had a precursor to viral infection (28.6%), and two developed the disease after vaccination (9.5%). Regarding immune comorbidities, one case was accompanied by myasthenia gravis, while another case was accompanied by inflammatory myopathy with the Ro52 antibody.

**FIGURE 4:**
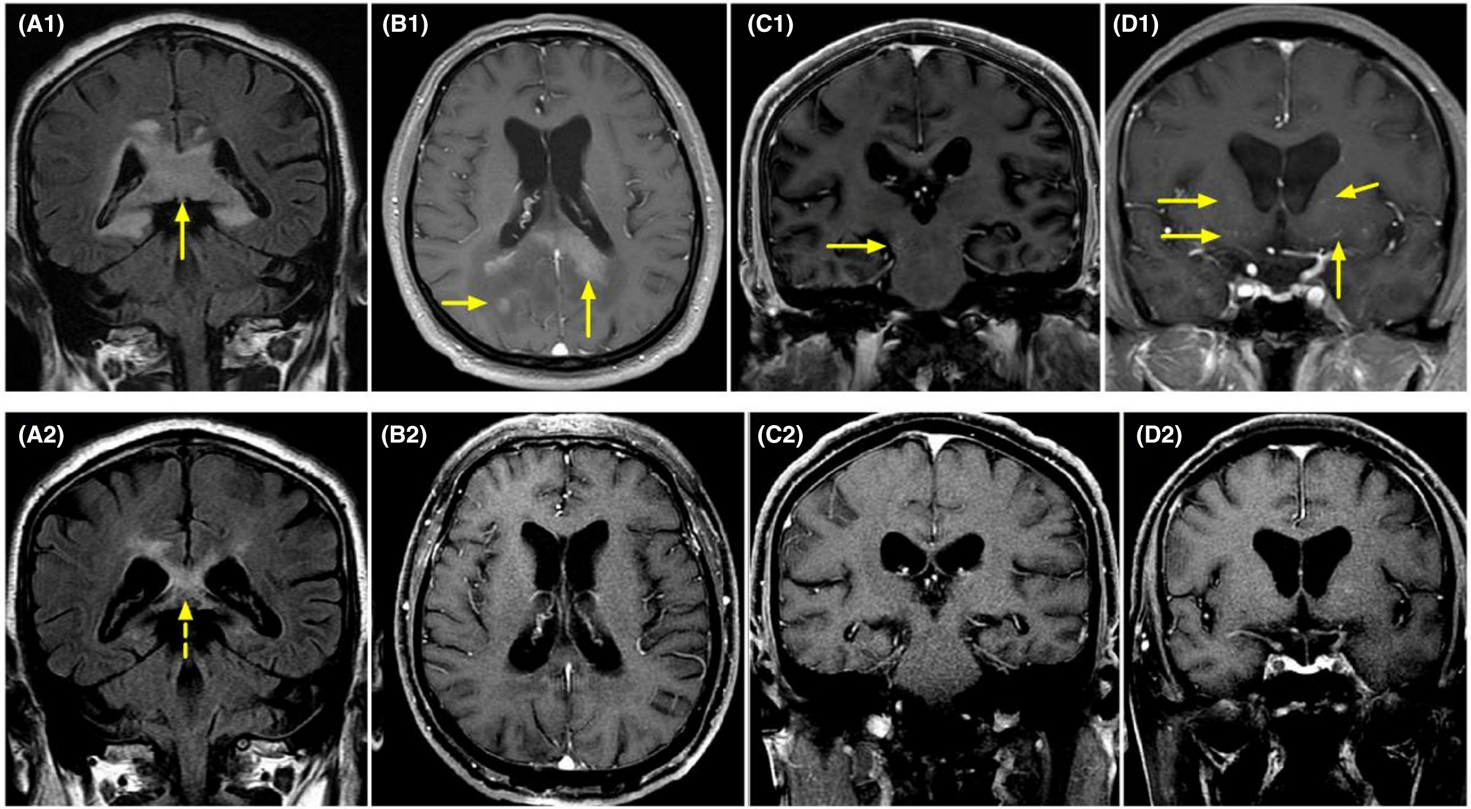
MRI features in a DNAP patient, Case 1. (A1) The T2‐abnormality is mainly located in the corpus callosum and has no obvious edema and mass effect. (A2) After intravenous methylprednisolone pulse therapy and mycophenolate mofetil treatment, the lesion is significantly reduced. (B1) Enhancement of lesions of the corpus callosum and occipital lobe. (C1) Outside the lesion, abnormal enhancement of the brainstem pia mater is observed (yellow arrow). (D1) Vascular enhancement is observed in the bilateral thalamus outside the lesion. (B2–D2) After treatment, no enhancement is observed in the parenchyma or pia mater.

On MRI (Figure 4), 10 patients had brain lesions (10/21, 47.6%), and three had spinal cord lesions (3/21, 14.3%). Twenty patients completed the EEG examinations, with eight (40.0%, 8/20) showing abnormal results. Of the 17 patients who completed electromyography examinations, 12 (70.6%, 12/17) were abnormal. Only five patients (23.8%, 5/21) had an increase in CSF white blood cell count, and seven patients (33.3%, 7/21) had an increase in protein levels. (Table S1).

### 3.5 Differences between DNAP, GFAP‐A and NMOSD

Differences between DNAP, GFAP‐A, and NMOSD are listed in Table S2. Compared with GFAP‐A, DNAP presented less fever (0/21 vs 3/7, *p* = 0.042) and urinary retention (0/21 vs 3/7, *p* = 0.042). Compared with NMOSD, DNAP presented less vision disturbances (2/21 vs 5/7, *p* = 0.018), less spinal cord lesions on MRI (4/20 vs 6/7, *p* = 0.024), less SEP (1/17 vs 5/7, *p* = 0.012) and VEP abnormality (2/17 vs 6/7, *p* = 0.009). There was no difference in other symptoms, history of cancer or infection, modified Rankin Scale (mRS), CSF, and electroencephalogram (Table S2).

### 3.6 Pathology of DNAP

**FIGURE 5:**
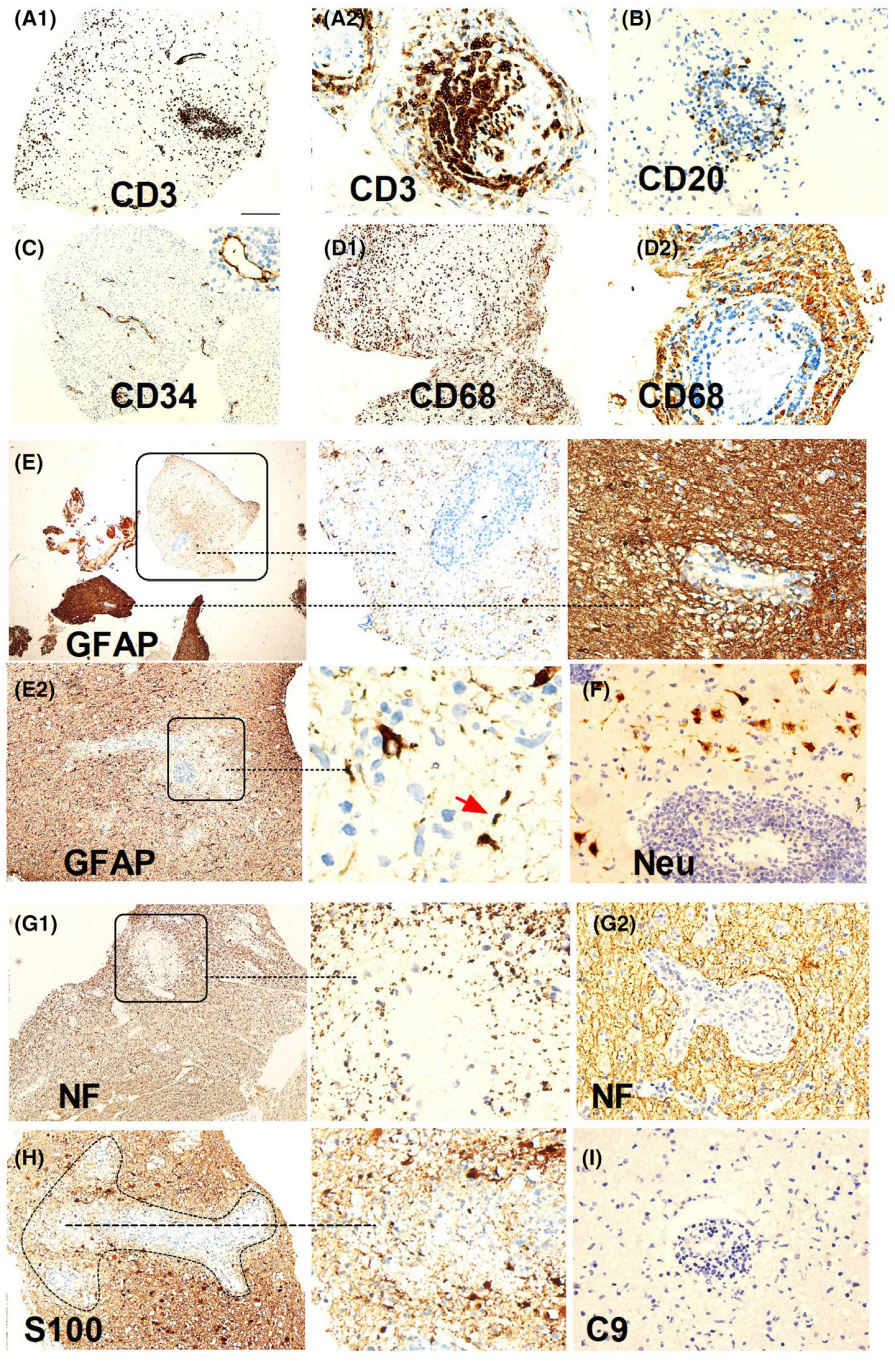
A pattern of pathology of double negative astrocytopathy (Case 1). (A1) Immunohistochemistry of the left frontal lobe biopsy showing infiltrates of prominent perivascular CD3+ T cells in the parenchyma. (A2) CD3+ T cells in the meninges around the vessels. (B) CD20+ B cells in the parenchyma. (C) Significantly increased microvessels (CD34) and inflammatory cells around the vessels. (D1, D2) CD68 staining highlights prominent microglial reactions and macrophage infiltration in the brain parenchyma (D1) and meninges (D2). (E) Compared with a relatively normal location, the lesion shows a severe loss of astrocytes (rectangular location). (E2) Local astrocyte loss (rectangular location) and debris (red arrows). (F) Relatively preserved neurons. (G1) Neurofilament loss is observed around the vessels compared with a relatively normal location (G2). (H) Local S100 loss. (I) No complementary deposition is observed in the lesion.

**FIGURE 6:**
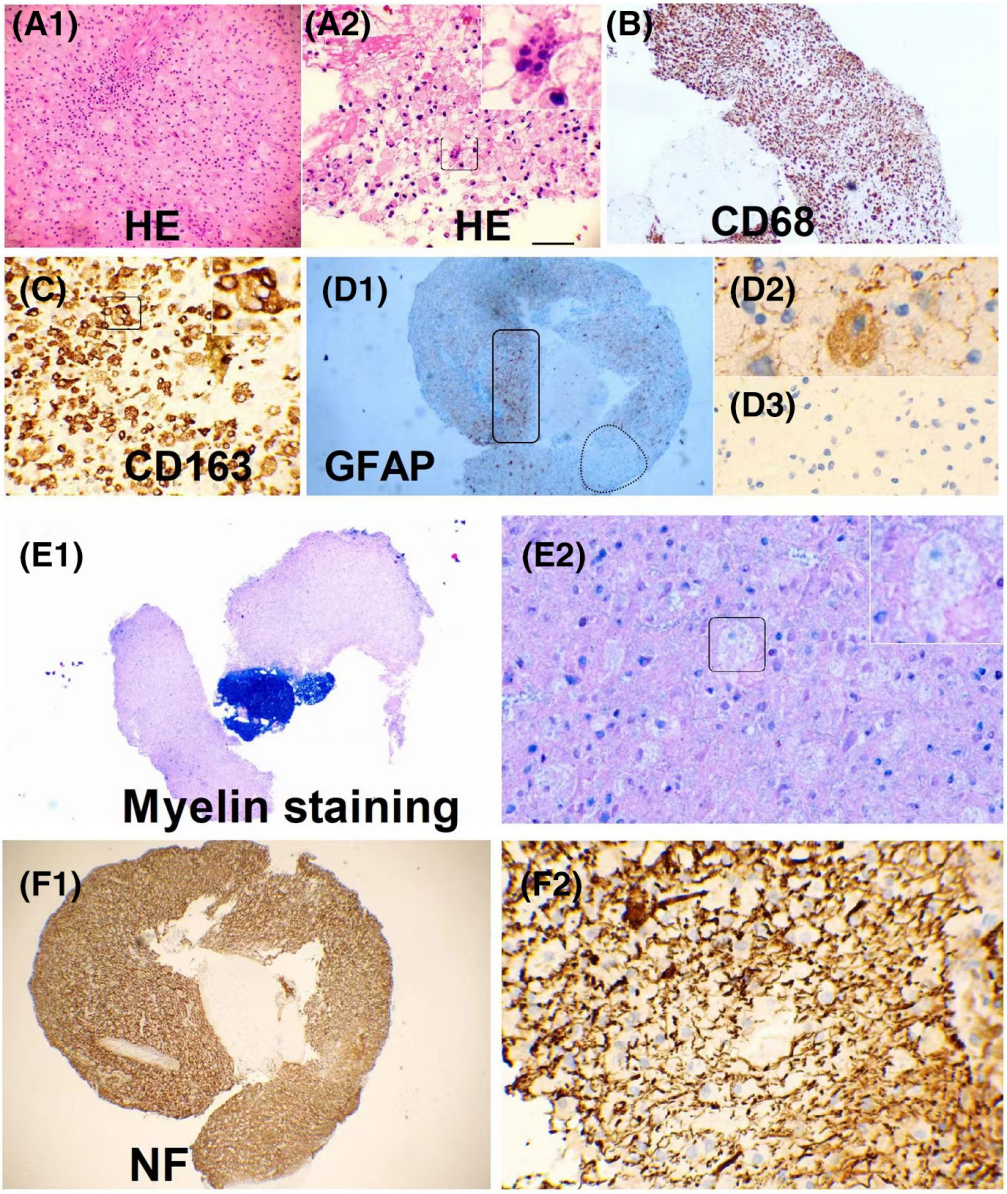
Another pattern of pathology of double negative astrocytopathy (Case 2). (A1) Right occipital lobe white matter lesion with perivenous inflammation and prominent tissue proliferation (hematoxylin and eosin staining). (A2) Higher magnification view of A1 reveals macrophages with several intracellular lymphocytes. (B) CD68 expression highlights prominent microglial reactions and macrophage infiltration. (C) CD163 expression highlights prominent microglial reactions and several intracellular lymphocytes. (D1) GFAP staining reveals increased immunoreactive astrocytes around the lymphocyte vascular sheath (rectangular location) and loss of astrocytes (oval location). (D2) Higher magnification view reveals hypertrophic astrocytes. (D3) Local loss of astrocytes. (E1) LFB/PAS staining reveals myelin loss. (E2) High‐magnification image showing macrophages with intracellular LFB‐positive debris. (F1, F2) Relatively preserved neurofilaments.

Two patients with suspected intracranial masses underwent stereotactic brain biopsy, both of who were found to have inflammation and demyelination. In one case (Figure 5), there was severe loss of astrocytes and loss of NF and S100 around the vessels, while in another case (Figure 6), there was prominent tissue proliferation and hypertrophied astrocytes with a local loss of astrocytes. Peripheral nerve biopsies were conducted in three patients, revealing a mild reduction in myelinated fibers, partial axial damage, and inflammatory cells around the vessels (Figure S1). Muscle biopsies were conducted in three cases, suggesting inflammatory myopathy in one case and secondary neurogenic injury in two cases, in one of which idiopathic inflammatory diseases could not be ruled out (Figure S2).

### 3.7 Treatment and outcome of DNAP

Of the 21 patients, 18 accepted immunotherapy, including steroids, immunoglobulin, immunoadsorption, rituximab, ofatumumab, and mycophenolate mofetil. The duration of each treatment is detailed in Table S1. Symptoms improved in those patients, except for one with an ALS‐like disorder (1/18, 5.6%).

## 4 DISCUSSION

It is challenging to define autoimmune astrocytopathy as negative for both AQP4‐IgG and GFAP‐IgG (DNAP). We referred to previous studies on NMOSD and GFAP‐A to establish our current definition. First, the presence of astrocyte antibodies implies autoimmune targeting of astrocytes. DNAP, similar to AQP4‐IgG and GFAP‐IgG, exhibited a pattern of astrocytopathy on TBA (Figure 2). Second, elevated GFAP levels in the CSF suggested the presence of injured astrocytes. According to previous studies, the release of GFAP in the CSF results from astrocyte injury and is associated with astrocytopathy in GFAP‐A and NMOSD.^1^, ^3^, ^10^, ^11^, ^12^, ^24^, ^25^ This study implies a connection between astrocytopathy and autoimmunity and high levels of GFAP linked to astrocytic lysis in pathology, with levels decreasing after immunotherapy. Furthermore, DNAP pathology revealed astrocytopathy, with similar findings to NMOSD and GFAP‐A pathological studies, including astrocytic lysis, microglial reactions, and hypertrophic.^1^, ^2^, ^5^, ^19^, ^26^

In terms of clinical manifestations or CSF features, DNAP is a group of diseases different from GFAP‐A and NMOSD. Similar to previous studies on GFAP‐A rather than on NMOSD, DNAP had a late onset (median age, 56 years) and did not exhibit a significant sex bias.^19^, ^27^, ^28^, ^29^ The clinical manifestations of DNAP were heterogenous, including encephalopathy, dyskinesia, mental symptoms, ataxia, and disorders of spinal cord, optic nerves and peripheral nerves. But some characteristic presentations of GFAP‐A and NMOSD were rare in DNAP, including fever, urinary retention, vision disturbances with abnormal VEP, and spinal cord lesions with abnormal SEP. In the CSF, although there was no statistic difference between DNAP, GFAP‐A, and NMOSD, which might be limited by the sample sizes of GFAP‐A and NMOSD, DNAP appears less severe clinically than GFAP‐A or NMOSD. CSF cytology results of DNAP were often normal, except for elevated blood counts in one case (Case 7), potentially due to herpes simplex virus infection. Protein, glucose, and chloride levels in the CSF of DNAP were relatively normal, compared with those in previous GFAP‐A studies where increased protein levels and decreased glucose and chloride levels were reported.^19^, ^27^, ^28^ DNAP appears less astrocyte injury than GFAP‐A or NMOSD, supported by the lower median GFAP levels in DNAP despite no significant statistical difference. As for the neuronal biomarker, the NFL levels in DNAP patients were lower than those in GFAP‐A patients (no statistical difference) but similar to those in NMOSD.

The pathology shed some light on these distinctions. We found two patterns in DNAP. In the brain biopsy of a patient with acute onset (Case 1), perivascular loss of neurofilament (NF) was observed, consistent with elevated NFL levels in the CSF, severe astrocyte loss, and relatively preserved neurons (Figure 5). Case 2 (Figure 6) showing pathology of neurodegeneration, lytic and sublytic astrocytes, loss of myelin, and relatively preserved NF were observed, aligning with the low NFL levels in the CSF. Compared with them, our prior pathological investigations have demonstrated that GFAP‐A is often accompanied by neuronal injury.^5^, ^19^, ^30^ This provides a plausible explanation for why, in the present study, neurofilament light chain (NFL) levels in GFAP‐A patients seemingly surpass those in DNAP patients. However, above results are still hard to explained because these observations may have been confounded by the heterogeneity of DNAP, which could be attributed to autoimmunity targeting different antigens, different phases of DNAP, and/or therefore different pathological findings.

It is still difficult to establish a uniform diagnosis of DNAP because the disease may be caused by autoimmune responses targeting different antigens. Most of our patients met the criteria of autoimmune encephalitis based on clinical phenotypes, ancillary tests, and biomarkers of neuronal damage.^31^ Although the antigens are still unclear, such unknown astrocyte antibodies may account for some autoantibody‐negative but probable autoimmune encephalitis reported in previous studies due to the absence or limited use of TBA.^32^ For instance, case 2 presented with impaired cognition, seizures, abnormal signals in the hippocampus on MRI, and abnormalities in EEG, which improved after immunotherapy, meeting the criteria for autoantibody‐negative AE.^31^ Therefore, a developmental perspective is required to identify more accurate biomarkers for antibody‐negative AE.

Interestingly, in the differential diagnosis, DNAP was required to exclude tumors. The two patients who underwent stereotactic brain biopsy were suspected to have intracranial masses on MRI (especially Case 1, Figure 4) but were diagnosed with intracranial demyelinating pseudotumors post‐biopsy. Despite the large intracranial lesions on imaging, the mass effect on surrounding areas of brain tissue or compressed structures was not significant. Additionally, they also presented with peripheral neuropathy and demyelination on EMG. Moreover, perivascular enhancement was observed, similar to that observed in the specific imaging of GFAP‐A (Figure 4). Due to limited knowledge of the disease, even with the positive astrocyte antibodies, tumors could not be excluded, warranting a brain biopsy. After routine immunotherapy with steroids and intravenous immunoglobulin, the patients showed significant improvement in clinical manifestations as well as imaging findings, along with a decline in the astrocyte biomarker, GFAP. Hence, after the exclusion of tumors and infections, early treatment with NMOSD or GFAP‐A is recommended for patients who are positive for astrocyte antibodies in the serum and CSF, particularly those with positive oligoclonal bands.

This study had some limitations. Firstly, there is the possibility of false negativity in the CBA for AQP4 antibodies in DNAP. Recently, an enzyme immunodot assay, developed for the detection of AQP4‐IgG, has been considered to have high sensitivity and specificity.^33^ However, in our 21 cases of DNAP, classical manifestations of NMOSD were not observed. Although their manifestations were similar to those of GFAP‐A, based on our experience, the staining patterns, such as staining within the Virchow–Robin space and CBA (Figure 2), were distinguishable from those of GFAP‐IgG.^9^, ^19^ Cross‐validation would enhance the validity of these findings. More importantly, the detection of astrocyte antibodies in the present study was based on TBA; therefore, the antigens targeted by these antibodies remain unknown. It was unclear whether there was a mixture of different antibodies. In the future, it will be important to confirm the target antigen using co‐immunoprecipitation, mass spectrometry, and CBA. Secondly, there are currently no available reference ranges for GFAP and NFL levels in the CSF of a normal population. Patients with functional neurological disorders who underwent CSF testing were not representative of the normal population; however, the results were similar to those of healthy controls in previous studies.^17^, ^34^ Besides, there was no difference between the NMOSD and control groups, which is inconsistent with previous studies and might raise questions about the NFL levels in NMOSD patients in the present study.^17^, ^34^ The result may have been confounded by the small sample size. Normal NFL levels with increased levels of GFAP were consistent with type 5 lesions described by Misu et al.^35^ where extensive astrocyte loss was typically observed in the absence of apparent axonal loss. Furthermore, the site of the peripheral nerve biopsy of the patient is the peroneal nerve (Case 17, Figure S1), the sensory nerve endings without the composition of astrocytes. Inflammation may not reflect autoimmunity‐targeting astrocytes. One possibility is that the damage could result from autoimmunity targeting antigens shared by Schwann cells and astrocytes, although they ontogenetically diverge from one another at an early developmental stage.^36^, ^37^ In Case 17, the autoantigen was confirmed as vimentin, which was confirmed to be expressed on Schwann cells.^38^, ^39^ What's more, inflammation may be caused by the accompanying overlap of immune responses. For example, in the present study, Case 12 had myasthenia gravis, and Case 11 (Figure S2) was positive for the Ro‐52 antibody, associated with a broad spectrum of autoimmune diseases, such as systemic lupus erythematosus, Sjogren's syndrome, and inflammatory myositis.^40^, ^41^ Lastly, in this study, brain pathology biopsy was not comprehensive. The samples were too small to apply additional indicators such as AQP4 expression and complement deposition. However, astrocytolysis and degeneration confirmed astrocytopathy.

In conclusion, other than GFAP‐IgG and AQP4‐IgG, unknown astrocyte antibodies have yet to be confirmed. The clinical manifestations of DNAP are highly heterogeneous, but changes in pathology and CSF biomarkers are consistent with astrocytopathy. These diseases are effectively treated with immunotherapy, with improvements in biomarkers and clinical presentation after treatment. Therefore, DNAP should be studied further in the future.

## AUTHOR CONTRIBUTIONS

Y‐ML accepts full responsibility for the work and the conduct of the study, and controlled the decision to publish. Conception and design of study: Y‐MLL. Acquisition of data: P‐HL, J‐JY, C‐CF, S‐FZ, CL, X‐LY, W‐DL, G‐YL, JY, Y‐HY, LH, SL, DL, B‐YL, Z‐FM, CG. Analysis of data: Y‐ML and P‐HL. Drafting a significant portion of the manuscript and figures: Y‐ML and P‐HL. Drafting parts of figures: C‐CF.

## FUNDING INFORMATION

This study was supported by Guangdong Basic and Applied Basic Research Foundation (2023A1515010225, 2022A1515110143), Multi‐center Project of The Second Affiliated Hospital of Guangzhou Medical University (2022‐LCYJ‐YYDZX‐04).

## CONFLICT OF INTEREST STATEMENT

The authors declare that no competing interests exist.

## Supporting information


Figure S1.

Table S1.

Table S2.

## Data Availability

The data that support the findings of this study are available from the corresponding author upon reasonable request.
