# Supplementary material for: Autoimmune astrocytopathy double negative for AQP4‐IgG and GFAP‐IgG: Retrospective research of clinical practice, biomarkers, and pathology
Source: CNS Neurosci Ther. 2024 Sep 15;30(9):e70042. doi: 10.1111/cns.70042 (PMC11402789; doi:10.1111/cns.70042)
Supplement: Supplementary file 1 — Figure S1. [file CNS-30-e70042-s003.docx]

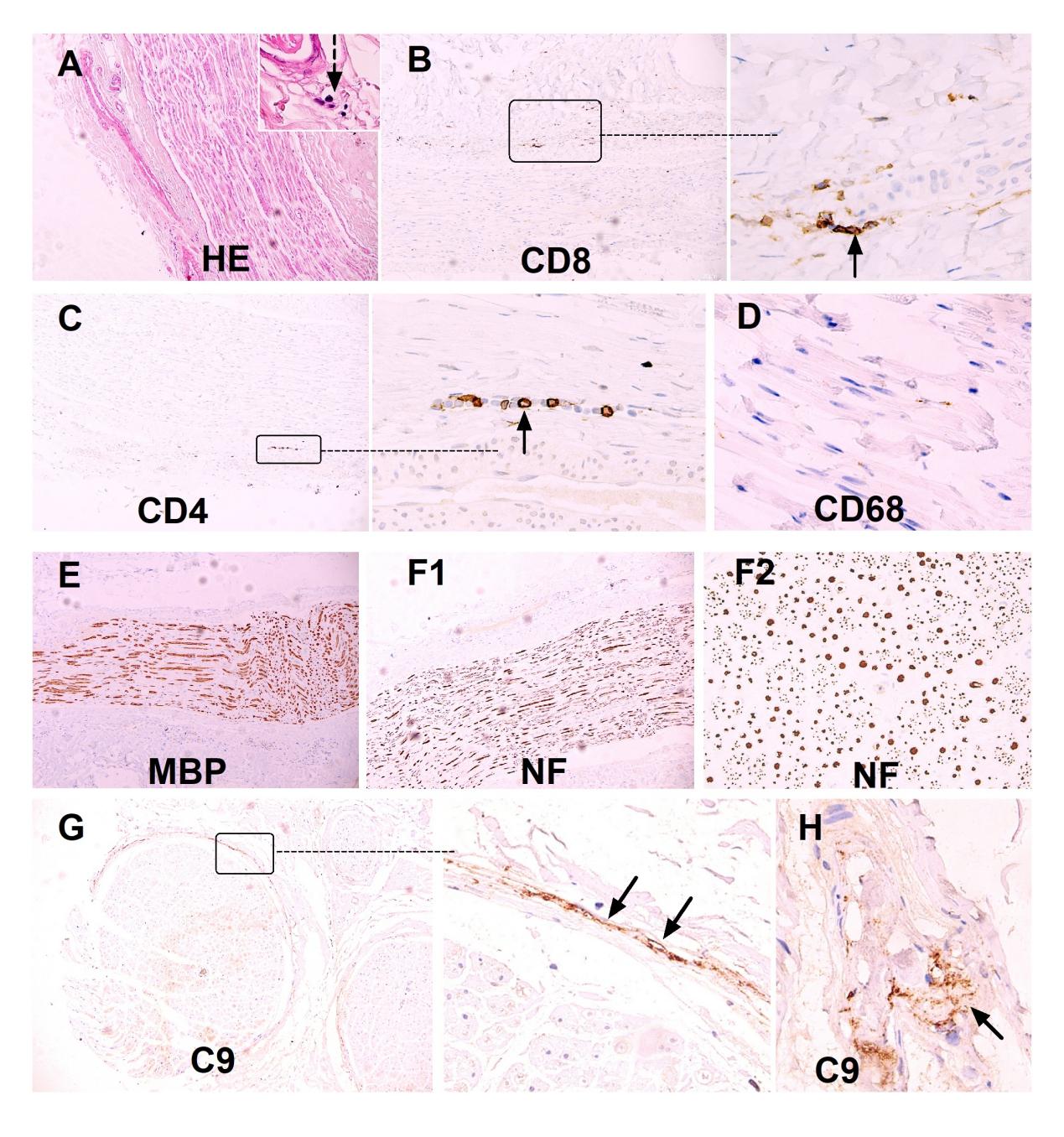


**Supplement Figure 1 Pathological changes of superficial peroneal nerve in a DNAP patient (Case 17) with ALS-Liked disorder**

A1: H&E stain showed inflammation cells around the wall of the nerve adventitia and blood vessels. B: Immunohistochemistry shows infiltrates of perivascular CD8 + T-cells. C: CD4 + T-cells. D: CD68 was negative. E, F1,F2: relatively normal MBP, NF. G,H: Complement deposition found around the wall of the nerve adventitia(G) and blood vessels(H).


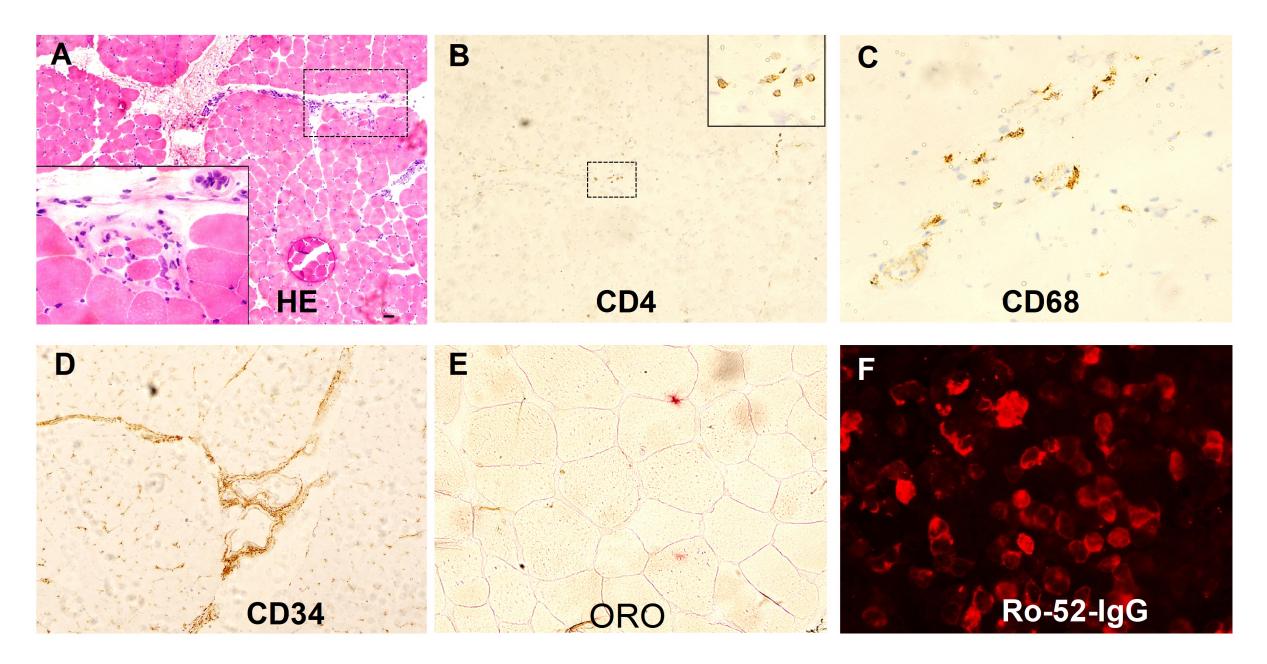


**Supplement Figure 2 Pathological changes of muscle and auto-antibody in a DNAP patient (Case 11) with neurological and muscular disorders**

A1: H&E stain showed scattered infiltration of inflammatory cells within the fascia and muscle fiber gaps. B: Immunohistochemistry shows CD4 + T-cells. C: Immunohistochemistry shows infiltrates of perivascular CD68 + cells. D: Significantly increased microvessels (CD34) E: Mild lipid deposition scattered within muscle fibers (ORO). F: Ro-52 antibody was positive by cell-based assay.


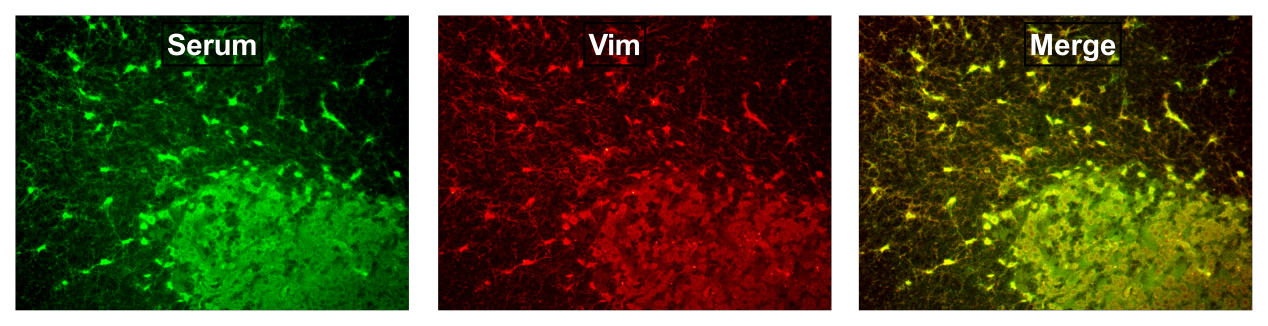


**Supplement Figure 3 A higher magnification to reveal a staining pattern that patient IgG (green) and commercial vimentin-IgG (Vim, red) colocalize in neurons (the bottom right) and astrocytes**
