# Supplementary material for: Autoimmune astrocytopathy double negative for AQP4‐IgG and GFAP‐IgG: Retrospective research of clinical practice, biomarkers, and pathology
Source: CNS Neurosci Ther. 2024 Sep 15;30(9):e70042. doi: 10.1111/cns.70042 (PMC11402789; doi:10.1111/cns.70042)
Supplement: Supplementary file 2 — Table S1. [file CNS-30-e70042-s002.docx]

**Supplement Table 1 Clinical features of patients with DNAP**

| No. | Duration/Relapse | Phenotypes | Main clinical manifestation | CSF | | | | | Infection/Cancer history | Cell targeted by antibodies | Antibody titers (Serum/CSF) | GFAP/NFL  (pg/ml) | Features of MRI | Electromyography/electroencephalogram | Immunotherapy, prognosis (mRS at onset / mRS at follow-up) |
| --- | --- | --- | --- | --- | --- | --- | --- | --- | --- | --- | --- | --- | --- | --- | --- |
|  |  |  |  | Pressure (mmH2O) | WBC /mm3 | Protein (mg/L) | Glucose (mmol/L) | Chloride (mmol/L) |  |  |  |  |  |  |  |
| 1 | 3 months/No | Encephalitis, peripheral neuropathy | Decreased memory and limbs weakness | 150 | 1 | 956 | 3.7 | 114 | No | Astrocytes and neurofilament | Negative/1:10 | 2443/549 | Enhancement in bilateral corpus callosum and occipital lobes | EEG:Increased diffuse waves; EMG: symmetrical peripheral neuropathy of limbs, involving motor and sensation, mainly demyelinating and secondary axonal degeneration. | Steriod (3 months) and MMF (1 month); 5/3 |
| 2 | 3 years/Yes | Encephalomyelitis | Epilepsy, impaired cognition and limb weakness | 150 | 1 | 327 | 3.3 | 119 | Suspected lung cancer | Astrocytes | 1:450/1:1 | 2892/621; | T2: Abnormal high signals in left frontal lobe, right hippocampus and cervical spinal cord | EEG: Left frontal lobe and bilateral temporal lobe spikes; EMG: right ulnar nerve and median nerve injury, demyelination, and abnormal SSR. | Steriod (3 years) and MMF (2 weeks), rituximab (2 times),; 2/0 |
| 3 | 1 month/No | Encephalitis | Consciousness disorders, seizures, dizziness | 170 | 0 | 261 | 3.0 | 124 | No | Astrocytes and neurofilament | 1:3200/Negative | 1659/348 | T2: Bilateral hippocampal abnormal high signals, cerebral atrophy | NA | Steriod (1 week); 4/1 |
| 4 | 1 months/No | Encephalitis | Abnormal behavior, drowsiness, headache, nausea | 135 | 1 | 143 | 4.6 | 130 | COVID-19 infection | Astrocytes | 1:1000/Negative | 1005/162 | Normal | EEG: mild to moderate abnormality; EMG: abnormal SSR. | Steriod (1 week); 2/0 |
| 5 | 1 month/No | Encephalitis | Behavioral abnormalities, decreased memory, and blurred consciousness | 220 | 28 | 652 | 3.2 | 109 | Herpes simplex virus infection | Astrocytes | 1:1000/1:3.2 | 13214/1358 | T2: Abnormal high signals in the right temporal lobe. | EEG: severe abnormality with slow spike wave release; EMG: NA. | IVMP (9 days), IVIG (3 days); 4/0 |
| 6 | 15 years/Yes | Encephalitis | Epilepsy,behavioral abnormalities, | 320 | 1 | 100 | 3.6 | 116 | No | Astrocytes | 1:320/Negative | 2376/214 | Bilateral temporal abnormalities | EEG: severe abnormality, sharp waves, and slow waves; EMG: NA. | Steroid (13 weeks); ,3/1 |
| 7 | 1 month/No | Encephalitis | Epilepsy and blurred consciousness | 190 | 12 | 1146 | 3.2 | 125 | No | Astrocytes | 1:320/1:32 | 776/325 | T2: Abnormal high signals in the subcortical area of the right temporal lobe | EEG: moderately abnormal; EMG: normal. | Steroid (2 weeks); 3/0 |
| 8 | 1 year/Yes | Encephalitis | Memory decline, sleep disorders | 150 | 6 | 357 | 3.7 | 121 | No | Astrocytes | 1:320/Negative | 1401/344 | T2: Multiple white matter lesions | EEG/EMG: normal. | No immunotherapy; 2/2 |
| 9 | 2 years/NO | Brainstem encephalitis | Dizziness and ataxia | 290 | 4 | 352 | 3.7 | 124 | No | Astrocytes and neurons | 1:3200/1:3.2 | 2212/258 | T2: Brainstem abnormality | EEG: severe abnormality, mainly slow wave; EMG: normal . | Steroid (12 weeks); 1/0 |
| 10 | 14 months/No | Peripheral neuropathy, optic neuritis, myelitis | Limb numbness, weakness, and decreased vision | 140 | 8 | 4490 | 3.9 | 128 | Suspected lung cancer | Astrocytes and neurons | Negative/1:10 | 1159/432 | T2: Lesions at the C4-6 levels of spinal cord | EEG: normal; EMG: severe peripheral nerve damage, involving motor and sensory fibers, coexisting with demyelination and axonal injury; VEP abnormality. | Steroid (8 months), Immunoadsorption (2 times) and MMF (1 week); 5/3 |
| 11 | 3 months/No | Movement disorder, peripheral neuropathy, myositis | Tremor and limb weakness | 185 | 1 | 246 | 4.3 | 126 | COVID-19 infection | Astrocytes | 1:10000/1:100 | 1913/374 | Non-specific lesions | EEG: NA; EMG: abnormality in the right ulnar nerve and SSR; BAEP abnormality. | Steroid (3 months), IVIG (1 day), IVMP (5 days) and immunoadsorption (3 times); 3/2 |
| 12 | 2 years/No | Movement disorder and myasthenia | Unstable walking, confusion, and bulbar paralysis | 90 | 2 | 1048 | 3.8 | 130 | No | Astrocytes | 1:10000/1:32 | 4010/746 | NA, but CT suggested mass in pineal body and white matter lesions. | NA | Steroid (2 weeks), IVIG (5 days), IVMP (5 days); 5/4 |
| 13 | 2 years/No | Movement disorder | Slowness of movement, numbness in lower extremity and sacrococcygeal regions | 100 | 2 | 234 | 4.0 | 122 | No | Astrocytes | 1:10/1:10 | 8496/358 | Enlarged ventricles and cerebral atrophy | EEG: normal; EMG: SSR abnormality. | No immunotherapy; 4/3 |
| 14 | 2 months/No | Movement disorder | Head involuntary movement and lower extremity numbness and weakness | 140 | 2 | 272 | 4.2 | 123 | COVID-19 infection | Astrocytes | 1:320/1:10 | 2548/334 | Non-specific lesions | Normal | Steroid (1 week); 1/0 |
| 15 | 3 months/No | Myeloradiculitis | lower extremity pain and weakness | 125 | 9 | 339 | 3.1 | 117 | herpes simplex virus history | Astrocytes | 1:10000/Negative | 1583/735; | Abnormal meningeal enhancement | EEG: NA; EMG: SSR abnormality. | Steroid (2 months), IVIG (5 days); 4/1 |
| 16 | 7 months/No | Ataxia | Unstable walking and bulbar paralysis | 185 | 0 | 168 | 4.0 | 122 | No | Astrocytes and neurons | 1:3200/1:000 | 1967/1982 | Non-specific lesions | EEG: NA; EMG: SSR abnormality | Steroid (3 months); 4/2 |
| 17 | 27 months/Yes | ALS like disease | Limbs weakness and atrophy | 170 | 4 | 398 | 3.1 | 122 | Post-vaccination | Astrocytes and neurons | 1:10000/1:1 | 1930/1667 | Non-specific lesions | EEG: NA; EMG: widespread motor nerve involvement. | Steroid (17 weeks), IVMP (5 days), MMF (1 week), IVIG (3 days), rituximab (2 times), immunoadsorption (6 times) ; 4/4 |
| 18 | 2 years/No | ALS like disease | Limbs weakness and atrophy | 50 | 2 | 687 | 4.6 | 124 | Post-vaccination | Astrocytes | 1:1000/Negative | 1938/1749 | Non-specific lesions | EEG: normal; EMG: widespread motor nerve involvement, abnormal myelin conduction and SSR; VEP abnormality. | No immunotherapy; 3/3 |
| 19 | 6 years/Yes | Myelitis | Limb numbness and weakness | 80 | 11 | 222 | 2.8 | 125 | No | Astrocytes | Negative/1:3.2 | 6277/2334; | Abnormal enhancement at the C1-3 levels of spinal cord | EEG: normal; EMG: normal | Steroid (6 years), rituximab (2 times); 3/1 |
| 20 | 6 months/Yes | ALS like disease | Limb numbness, atrophy and weakness | 220 | 1 | 1016 | 3.3 | 124 | Upper respiratory tract infection | Astrocytes and neurofilament | 1:100/1:1 | 2447/2274; | Non-specific lesions | EEG: normal; EMG: neurogenic damage in the medullary, cervical, and thoracic segment of spinal cord (anterior horn or anterior root), and bilateral ulnar nerve elbow damage | Steroid (6 months), ofatumumab (2 months) and immunoadsorption (4 times); 3/1；ALS 36/40 |
| 21 | 2 months/ Yes | Myelitis | Limb numbness and weakness | 135 | 3 | 275 | 4.8 | 118 | COVID-19 infection | Astrocytes | 1:1/Negative | 1620/2463; | T2: Lesions at the C2-4 and T1-4 levels of spinal cord | EEG: moderate abnormality; EMG: abnormal SSR in limbs and abnormal SEP above T12. | Steroid (2 months), MMP (1 week) and immunoadsorption (2 times); 3/0 |

F: female; M: male; MRI: magnetic resonance imaging; ALS: amyotrophic lateral sclerosis; EEG: electromyography; EMG: electroencephalogram; SSR: sympathetic skin response; VEP: visually-evoked potential; BAEP: brain stem auditory evoked potential; SEP: somatosensory evoked potentials; mRS: modified Rankin Scale; MMF: mycophenolate mofetil; IVMP: intravenous high-dose methylprednisolone; IVIG: intravenous immunoglobulin
