# Supplementary material for: Autoimmune astrocytopathy double negative for AQP4‐IgG and GFAP‐IgG: Retrospective research of clinical practice, biomarkers, and pathology
Source: CNS Neurosci Ther. 2024 Sep 15;30(9):e70042. doi: 10.1111/cns.70042 (PMC11402789; doi:10.1111/cns.70042)
Supplement: Supplementary file 3 — Table S2. [file CNS-30-e70042-s001.docx]

**Supplement Table 2 Differences between DNAP, GFAP-A and NMOSD**

|  | DNAP (n=21) | GFAP-A (n=7) | NMOSD (n=7) | DNAP vs GFAP-A | DNAP vs NMOSD |
| --- | --- | --- | --- | --- | --- |
| Age | 52.0±21.35 | 59.25±16.66 | 42.71±12.07 | 0.368^*^ | |
| Female | 11.0/21.0, 52.38% | 2.0/7.0, 28.57% | 7.0/7.0, 100.0% | >0.999 | 0.207 |
| Fever | 0/21.0, 0.0% | 3.0/7.0, 42.86% | 0/7.0, 0.0% | 0.042 | Unavailable |
| Limb weakness | 11.0/21.0, 52.38% | 3.0/7.0, 42.86% | 5.0/7.0, 71.43% | >0.999 | >0.999 |
| Decreased cognitive function | 5.0/21.0, 23.81% | 0/7.0, 0.0% | 0/7.0, 0.0% | >0.999 | >0.999 |
| Disorders of consciousness | 4.0/21.0, 19.05% | 1.0/7.0, 14.29% | 0/7.0, 0.0% | >0.999 | >0.999 |
| Behavioral abnormalities | 3.0/21.0, 14.29% | 1.0/7.0, 14.29% | 0/7.0, 0.0% | >0.999 | >0.999 |
| Seizure | 5.0/21.0, 23.81% | 0/7.0, 0.0% | 0/7.0, 0.0% | >0.999 | >0.999 |
| Vision disturbances | 1.0/21.0, 9.52% | 1.0/7.0, 14.29% | 5.0/7.0, 71.43% | >0.999 | 0.003 |
| Urinary retention | 0/21.0, 0.0% | 3.0/7.0, 42.86% | 1.0/7.0, 14.29% | 0.042 | >0.999 |
| Post-Infection/Vaccination History | 9.0/21.0, 42.86% | 3.0/7.0, 42.86% | 1.0/7.0, 14.29% | >0.999 | >0.999 |
| Cancer | 2.0/21.0, 9.52% | 0/7.0, 0.0% | 0/7.0, 0.0% | >0.999 | >0.999 |
| mRS at admission | 3.0(1.0-5.0) | 4.5(2.0-5.0) | 4.0(2.0-5.0) | 0.370^*^ | |
| mRs at follow-up | 1.0(0.0-4.0) | 1.5(1.0-3.0) | 2.0(1.0-3.0) | 0.503^*^ | |
| Immunotherapy | 18.0/21.0, 85.71% | 7.0/7.0, 100% | 7.0/7.0, 100% | >0.999 | >0.999 |
| Improvement | 18.0/21.0, 85.71% | 7.0/7.0, 100% | 6.0/7.0, 85.71% | >0.999 | >0.999 |
| CSF pressure, mmH2O | 161.67±64.25 | 151.25±49.39 | 146.43±28.39 | 0.812^*^ | |
| CSF WBC, cells/mm^3^ | 2.0(0.0-28.0) | 13.0(1.0-36.0) | 5.0(1.0-58.0) | 0.153^*^ | |
| CSF protein, mg/L | 339.0(100.0-4490.0) | 862.0(413.0-3437.0) | 420.0(181.0-678.0) | 0.153^*^ | |
| CSF Glucose, mmol/L | 3.7(2.8-4.8) | 4.45(3.3-9.4) | 3.2(2.3-6.9) | 0.28^*^ | |
| CSF Chloride, mmol/L | 122.05±5.17 | 120.25±4.57 | 123.14±5.46 | 0.675^*^ | |
| Antibody positive in serum | 18.0/21.0, 85.71% | 4.0/7.0, 57.14% | 0/7.0, 0.0% | 0.861 | >0.999 |
| Antibody positive in CSF | 14.0/21.0, 66.67% | 7.0/7.0, 100% | 5.0/7.0, 71.43% | 0.624 | >0.999 |
| MRI Intracranial lesion | 9.0/20.0, 45.0% | 5.0/7.0, 71.43% | 4.0/7.0, 57.14% | >0.999 | >0.999 |
| MRI Spinal cord lesion | 4.0/20.0, 20.0% | 3.0/7.0, 42.86% | 6.0/7.0, 85.71% | >0.999 | 0.024 |
| Electroencephalogram | 8.0/15.0, 53.33% | 3.0/4.0, 75.0% | 5.0/7.0, 71.43% | >0.999 | >0.999 |
| Electromyography | 12.0/17.0, 70.59% | 3.0/7.0, 42.86% | 0/7.0, 0.0% | >0.999 | 0.867 |
| SSR abnormality | 8.0/17.0, 47.06% | 3.0/4.0, 75.0% | 3.0/7.0, 42.86% | >0.999 | >0.999 |
| SEP abnormality | 1.0/17.0, 5.88% | 2.0/4.0, 50.0% | 5.0/7.0, 71.43% | 0.42 | 0.012 |
| VEP abnormality | 2.0/17.0, 11.76% | 1.0/4.0, 25.0% | 6.0/7.0, 85.71% | >0.999 | 0.009 |

*There was no difference between the three group.

SSR: sympathetic skin response; VEP: visually-evoked potential; SEP: somatosensory evoked potentials; mRS: modified Rankin Scale.
